# Early Clinical Results of Perceval Sutureless Aortic Valve in 139 Patients: Freeman Experience

**DOI:** 10.21470/1678-9741-2017-0087

**Published:** 2018

**Authors:** Syed Saleem Mujtaba, Simon Ledingham, Asif Raza Shah, Stephen Clark, Thasee Pillay, Stephan Schueler

**Affiliations:** 1 Department of Cardiothoracic Surgery, Freeman Hospital, Freeman Road, United Kingdom of Great Britain and Northern Ireland.

**Keywords:** Aortic valve stenosis, Aortic Valve/Surgery, Bioprosthesis, Heart Valve Prosthesis, Heart Valve Prosthesis Implantation/Methods, Minimally Invasive Surgical Procedures

## Abstract

**Objective:**

The aim of this retrospective study is to evaluate the safety and performance of the Perceval sutureless valve in patients undergoing aortic valve replacement. We report the 30-day clinical outcomes of 139 patients.

**Methods:**

From January 2014 to December 2016, 139 patients underwent sutureless aortic valve replacement. Their operation notes, National Adult Cardiac Surgery Database and perioperative transoesophageal echocardiography findings were studied retrospectively.

**Results:**

Ninety-two patients underwent isolated aortic valve replacement (group A) with Perceval valve and 47 patients had combined procedures of aortic valve replacement and coronary artery bypass grafting (group B). The patients received a size S (n=23), M (n=39), L (n=42) or XL (n=35) prosthesis. Perceval valve was successfully implanted in 135 (97.1%) patients. Mean cross-clamping time and bypass time were 40 and 63 minutes for isolated cases, while 68 and 107 minutes for combined cases. Three (2.1%) patients died within 30 days. Four patients suffered stroke and 5 patients went into acute renal failure. Median intensive care unit and hospital stay was 2 and 8.5, respectively. Four valves were explanted due to significant paravalvular leak after surgery. Five patients had permanent pacemaker as a result of complete heart block and mean postoperative drainage was 295 mL for isolated case and 457 mL for combined cases. The mean gradient across Perceval valve was 12.5 mmHg while its effective orifice area was 1.5 cm^2^.

**Conclusion:**

Early postoperative results showed that Perceval valve is safe. Further follow up is needed to evaluate the long-term outcome with this bioprosthesis.

**Table:** 

Abbreviations, acronyms & symbols	
AVR	= Aortic valve replacement
CABG	= Coronary artery bypass grafting
CPB	= Cardiopulmonary bypass
EuroSCORE	= European System for Cardiac Operative Risk Evaluation
FEV1	= Forced expiratory volume in 1 second
ICU	= Intensive care unit
NACSA	= National Adult Cardiac Surgery Audit
NICOR	= National Institute for Cardiovascular Outcomes Research
PVL	= Paravalvular leak
SCTS	= Society for Cardiothoracic Surgeons
STS	= Society of Thoracic Surgeons
TAVI	= Transfemoral aortic valve implantation
TOE	= Transesophageal echocardiography

## INTRODUCTION

Severe aortic valve stenosis is a common cardiac disease among the elderly. Due to the increasing age of the patient population in the Western world, there has been an increase in the prevalence of patients with valvular heart disease eligible for aortic valve replacement (AVR)^[1]^. AVR is the treatment of choice for aortic valve stenosis when it is severe aortic stenosis (≤ 1 cm^2^/m^2^) and symptomatic, or asymptomatic with left ventricular dysfunction, or combined with other cardiac surgery procedure^[2]^. Given the increasing number of comorbidities and increasing patient age, a tendency has emerged to use biological valve implants, avoiding the need for long-term anticoagulation therapy.

Although transapical or transfemoral aortic valve implantations (TAVIs) have been introduced for high-risk patients ineligible for conventional AVR using cardiopulmonary bypass (CPB), it has its own limitations, *e.g*., significantly increased costs, inability to remove the calcified aortic valve, and the high incidence of paravalvular leak (PVL), pacemaker implantation and neurological events^[3]^.

In last few years sutureless aortic valve bioprostheses have been developed to facilitate surgical AVR, reduce aortic cross-clamping and CPB time and curtail risk of mortality and morbidity. Sutureless aortic bioprosthesis implantation is a feasible alternative for high-risk patients with aortic valve disease^[4]^. The Perceval valve (Sorin Group, Saluggia, Italy) is a bioprosthetic heart valve made of bovine pericardium that allows a fast implantation through a sutureless technique. The current study reports our single-centre experiences regarding the early outcomes of Perceval valve implantation.

## METHODS

The Perceval valve is a surgical bioprosthetic heart valve composed of glutaraldehyde-fixed bovine pericardium treated with homocysteic acid in order to remove the free aldehyde residues and prevent the calcification process. It is fixed in a metal cage made up of an alloy of nickel and titanium, known as nitinol. Currently, four sizes of the Perceval S aortic valve prosthesis are available: small - S (19-21 mm); medium - M (22-23 mm), large - L (24-25 mm) and extra-large XL (27 mm). Hence, Perceval S can be used for annulus sizes ranging from 19 to 27 mm.

We performed a retrospective analysis of a consecutive series of patients (n = 139) who underwent AVR with Perceval valve between January 2014 and December 2016. They were either isolated AVR (group A, n = 92) or combined with coronary artery bypass grafting (CABG, group B, n = 47). Inclusion criteria were severe aortic valve stenosis with indication of valve replacement, patients who agreed to participate in the clinical evaluation, patients wishing to have a tissue valve, no contraindication for Perceval valve, and signed informed consent. These patients were operated by six different surgeons. During the same period, 312 patients had AVR with conventional Perimount Magna biological valve by four different surgeons.

Patients with ascending aortic aneurysm or dissection, emergency intervention, acute endocarditis, congenital bicuspid aortic valve (Seivers type 0) or aortic annulus greater than 27 mm or less than 21 mm were excluded. The ratio between the diameter of the sinotubular junction and aortic annulus should not exceed 1.3 mm. Patients with known nickel hypersensitivity were also avoided.

The operative risk of these patients was estimated according to the logistic European System for Cardiac Operative Risk Evaluation (logistic EuroSCORE). In this study, we have used logistic EuroSCORE as we submit patient's risk assessment as logistic EuroSCORE to National Adult Cardiac Surgery Audit (NACSA) database and it is easier to retrieve in this form. The data used in this analysis were extracted from NACSA. The audit is managed by National Institute for Cardiovascular Outcomes Research (NICOR), with clinical direction and strategy provided by the Society for Cardiothoracic Surgeons (SCTS) and the Project Board.

### Surgical Technique

After a full median sternotomy, standard CPB was established by cannulation of the ascending aorta and the right atrium. The heart is vented through the right superior pulmonary vein. Antegrade cardioplegia is the method of choice at our institution for myocardial protection. Continuous carbon dioxide insufflation was used routinely after sternotomy until closure of the aortotomy. The ascending aorta was incised transversally 1.5 cm above the sinotubular junction in order to leave a free edge for closure of the aortotomy after implantation of the device. The aortic valve was removed, and the annulus was decalcified in the usual fashion. The aortic orifice was measured with the original sizer of the bioprosthesis.

Three 4-0 polypropylene guiding sutures were passed at the nadir of the aortic annulus. An appropriately sized prosthesis was collapsed in a side table and placed into the manufacturer's holder.

The three guiding sutures were passed through the three green holes arising from the annular ring of the prosthesis, which was consequently seated on the debrided annulus. Once the delivery system is in position, the stent is deployed by turning the release screw and leaving the valve in place. The delivery system and the guiding sutures are removed. The field was rinsed with warm saline, and the prosthesis was dilated at four atmospheres for 30 seconds. After closure of the aortotomy, transesophageal echocardiography (TOE) was performed to assess the correct implantation of the prosthesis and the presence of any PVL.

## RESULTS

The mean age of the 139 patients was 74.3 years (range 47-86 years); 21.6% of patients were ≥ 80 years old (Table 1). The procedure success rate was 97.1%. In four (2.8%) patients Perceval valve was explanted mainly due to severe PVL, erroneous sizing or malpositioning discovered during perioperative echocardiography. In each one of these cases, a different prosthetic valve was eventually implanted. Mean crossclamping time and bypass time were 40 and 63 minutes for isolated cases while 68 and 107 minutes for combined cases.

**Table 1:** Preoperative summary.

	Total n = 139n	%	
Gender (Male/Female)	65/74	47/53	
Procedure types			
Isolated aortic valve replacement (group A)	92	66	
AVR + CABG or other procedures (group B)	47	34	
Cigarette smoking history	83	60	
History of hypertension	106	76	
Renal disease at time of surgery	1	0.7	
History of pulmonary disease (*e.g.* COPD, asthma)	2	1.44	
History of neurological disease (*e.g*. TIA, CVA)	25	18	
Angina status pre-surgery			
0. No angina	49	35	
1. No limitation of physical activity	33	24	
2. Slight limitation of ordinary activity	29	21	
3. Marked limitation of ordinary physical activity	25	18	
4. Symptoms at rest or minimal activity	3	2	
Dyspnoea status pre-surgery			
1. No limitation of physical activity	12	8.7	
2. Slight limitation of ordinary physical activity	53	38	
3. Marked limitation of ordinary physical activity	71	51	
4. Symptoms at rest or minimal activity	3	2	
History of Diabetes mellitus	30	22	
Preoperative heart rhythm			
0. Sinus rhythm	109	78	
1. Atrial fibrillation/flutter	28	20	
2. Complete heart block/pacing	2	1.4	
Ejection fraction category			
1. Good (LVEF > 50%)	111	80	
2. Fair (LVEF 30-50%)	22	16	
3. Poor (LVEF < 30%)	6	4	
	**Range**	**Mean**	**Median**
Age of patients at time of procedure	47-86	74.3	75.5
Logistic EuroSCORE comparison	0.53-18.886	3.26	2.457
Height (cm)	140-185	162	162
Weight (kg)	40.3-158	79	74

AVR=aortic valve replacement; CABG=coronary artery bypass grafting; COPD=chronic obstructive pulmonary disease; CVA=cerebrovascular accident; EuroSCORE=European System for Cardiac Operative Risk Evaluation; LVEF=left ventricular ejection fraction; TIA=transient ischemic attack

Size S (annulus range 19-21 mm) was implanted in 23 (17%) of patients, size M (annulus range 21-23 mm) in 39 (28%), size L (annulus range 23-25 mm) in 42 (30%) and size XL (annulus range 25-27 mm), which was available since July 2012, was implanted in 35 (25%) of the eligible patients.

The mean length of intensive care unit (ICU) stay was 3.4 days (range 1-32 days, median 1 day) in isolated AVR while it was 6.8 days (range 1-93 days, median 3 days) in combined cases. Hospital stay was 11.7 days (median 8, range 4-48) for isolated cases while it was 18.4 days (median 9, 4-225) for combined cases (Tables 2 and 3).

**Table 2:** Postoperative summary.

	Group A n = 92			Group B n = 47		
	Range	Mean	Median	Range	Mean	Median
Cumulative cross-clamp time (min)	21-114	40	37	28-127	68	61
Cumulative bypass time	25-172	63	59	38-403	107	87
Postoperative blood loss at 12 hours	50-2000	295	225	50-1200	457	400
ICU stay in days	1 32	3.4	1	1 93	6.8	3
Reoperation for bleeding, tamponade or valvular problems	2 (1.4%)			1 (0.7%)		

ICU=intensive care unit

**Table 3:** Postoperative summary.

	n = 139	%
Reoperation for bleeding, tamponade or valvular problems	3	2.1%
Sternal wound infection	2/139	1.43%
New postoperative neurological dysfunction	4/139	2.8%
New HF/dialysis postoperatively	5/139	3.6%
Patient status at discharge (mortality)	3/139	2.1%
SIRS	21/139	15%
Arrhythmias		
None	88	63%
AF/flutter	46	33%
Permanent pacemaker	5	3.6%

AF=atrial fibrillation; HF=hemofiltration; SIRS=systemic inflammatory response syndrome

No cases of unanticipated adverse device effects, valve thrombosis, secondary valve dislodgement or valve-related haemolytic anaemia were reported. Five (3.6%) patients developed renal failure and were connected to continuous venovenous hemofiltration leading to recovery of renal function. Forty-six (33%) patients had postoperative atrial fibrillation, were treated with betablockers or amiodarone and returned to sinus rhythm. A total of 4 (2.8%) postoperative strokes occurred and 3 (2.1%) cases of early bleeding led to reoperation. The incidence rate of permanent pacemaker implantation was overall 3.6%.

The mean gradient across Perceval valve was found to be higher for the smaller and lower for the larger valves. It ranges from 6-18 mmHg and its mean value was 12.5 mmHg. Similarly, its effective orifice area varies according to the valve sizes but its mean value was 1.5 cm^2^.

In-hospital mortality was 2.1% (n=3). The first patient was a 70 years old man who had AVR and CABG. He was re-intubated for respiratory compromise on day 3 and aspirated during the procedure. He went into sepsis, renal failure and developed ischemic colon. Finally, he died on day 13. The next one was a 70 years old male, chronic smoker, severe chronic obstructive pulmonary disease, forced expiratory volume in 1 second (FEV1) 1.4 lit (53% predicted). He underwent uneventful surgery and ICU stay. On day 4, he had a run of ventricular tachycardia, followed by respiratory and cardiac arrest. He was intubated, resuscitated and chest was opened. There was no evidence of tamponade, at this stage he went into ventricular fibrillation and finally died despite all resuscitation attempts. The last one was a 70 years old, 47 kg frail woman with severely impaired left ventricle and severe aortic stenosis. She had uneventful surgery but died of low output syndrome and heart failure on day 14.

## DISCUSSION

Magovern & Cromie^[5]^ introduced the concept of sutureless aortic valve in the 1960s with the ball-cage-type mechanical valve for sutureless implantation. It had its own disadvantages, *e.g.* high incidence of PVLs, bulky size and not suitable for small annuli^[6]^, high incidence of thromboembolism (42%) and reoperation (16%)[6]. This valve continued to be used until 1980.

When aortic valve is replaced surgically, sutures are placed into the annulus and then through the sewing cuff of the prosthetic valve. While at sutureless aortic valve, it obviates the need to put sutures to fix the valve, and thus make the procedure faster, cross-clamp and CPB times shorter, both are independent predictors of 30 day postoperative mortality after adult cardiac surgery^[7]^.

High-risk patients, particularly those undergoing prolonged concomitant surgery and redo surgery, could benefit from reduced length of time of the implantation by avoiding the need to use sutures to secure the bioprosthesis within the aortic annulus. Shrestha et al.^[8]^ also confirmed the safety and efficacy of the sutureless aortic valve in patients requiring concomitant procedures. This is important as, according to the Society of Thoracic Surgeons (STS) database, the proportion of candidates requiring concomitant CABG has risen from 5 to 25% over the past 20 years.

The effective orifice area of the valve is more for any given valve size as there is no ring for valve anchorage. This would be particularly beneficial for patients with small aortic roots where risk of patient prosthesis mismatch is high^[9]^.

Sutureless valves are also advantageous in minimally invasive AVR as it is technically difficult to put annular sutures in such cases because of the limitation of working space. Sutureless valves obviate this technical difficulty.

The durability of the Perceval valve is also an issue. Englberger et al.^[10]^ presented the longest follow-up study for a sutureless bioprosthesis (5-year follow-up) and suggested that sutureless valves become an option for all patients with indicated biological AVR.

The PARTNER trial showed a significantly higher incidence of PVL following TAVI than after surgical AVR at both 1 and 2 years^[11]^. PVL has been identified as an independent predictor of late mortality after TAVI^[12]^. D'Onofrio et al.^[13]^ showed that the incidence of PVL (at least mild) was higher in a transapical TAVI group compared with a sutureless bioprosthesis group (44.7% *vs.* 15.8%, respectively, *P*=0.001). Unlike TAVI, it is technically possible to perform repositioning and to exchange the sutureless valve intraoperatively if the result is unsatisfactory.

PVL can be the result of inadequate sizing, malpositioning or inappropriate decalcification of the annulus^[14]^. Recent evidence from TAVI trials has demonstrated a significant correlation between PVL and poorer outcomes. PVL was demonstrated to be a significant predictor of one-year mortality, even after multivariable adjustment.

Unlike TAVI and similarly to conventional AVR, the sutureless AVR approach involves excision of the calcified valve and prosthesis placement under direct visualization on a still heart, which may reduce the risk of misplacement and PVL^[15]^. Pollari et al.^[16]^ found shorter ICU stays, hospital stays, and intubation times in the sutureless group then in the stented group.

Even in cases requiring explantation of Perceval, the procedure was easy and the Perceval valve was removed without technical issues, as previously described^[17]^. Careful patient selection and echocardiographic assessment are crucial in choosing the proper size. Correct sizing of the valve is critical to minimize PVL and this should be performed with TOE and intraoperative sizing.

We have compared our perioperative and early postoperative results with other published series using sutureless valves (Table 4). Most of the authors have used Perceval valve except two who used Edwards Intuity and ATS 3f Enable valves. Our mean logistic EuroSCORE is less then all of the studies as we have least comorbidities. Our cross-clamp and bypass time for the isolated Perceval AVR is almost the same as for most of the studies but longer then Folliguet et al.^[14]^, Flameng et al.^[18]^, Cavalier trial^[19]^ and Pollari et al.^[16]^. We replaced 2.8% valves for severe PVL. This rate was 1.9%, 2.1%, 1.2%, 1.6% and 0.8% for Kocher et al.^[20]^, Martens et al.^[21]^, Santarpino et al.^[22]^, Shrestha et al.^[8]^ and Cavalier trial^[19]^, respectively. Folliguet et al.^[14]^ explanted 4.6% valves for severe PVL.

**Table 4:** Comparison of our results with other published series using sutureless valves.

Study	Type of valve	Mean logistic EuroSCORE	Cross-clamp time min Isolated AVR	Bypass time min Isolated AVR	Severe PV leak/replacedvalve N (%)	AV block N (%)	Mortality	Meangradient mmHg	Mean effective orifice area cm^2^
Our study (2018) n=139	Perceval S	3.26 (0.53-18.8)	40 (21-114)	63 (25-172)	4 (2.8%)	5 (3.6%)	3 (2.1%)	12.5 (6-18)	1.5
D'Onofrio^[13]^ (2012) n=51	Perceval S	14.2±8.1	44±17	69±26		2 (5.3%)	0	10.9±3.72	
Folliguet^[14]^ (2012) n=208	Perceval S	8.7±5.3	30.1±12.2	50.3±22.8	9 (4.3%)	16 (7.7%)	5 (2.4%)	10.4±4.3	1.4±0.4
Kocher^[20]^ (2013) n=146	Edwards Intuity	7.9±6.5	41.1±10.6	66.3±18.7	2 (1.9%) but after 30 days	10 (7.1%)	3 (2.1)	8.8±3.0	1.7±0.2
Martens^[21]^ (2011) n=140	ATS 3f Enable		58.1±25.1Including combined procedures	84.9±34.2Including combined procedures	3 (2.1%)		5 (3.6%)	10.24±4.2	1.75±0.45
Santarpino^[22]^ (2012) n=83	Perceval S	10±7.5	36±12.7	66±21	1 (1.2%)	3 (3.6%)	2 (2.4%)	13.4±2.8	
Flameng^[18]^ (2011) n=32	Perceval S	9.99	17 (12-34)	46(24-120)		1 (3.1%) 6-12 months after surgery	3 (9.4%) 6-12 months after surgery		1.5 (0.8-2.2)
Shrestha^[8]^ (2014) n=243	Perceval S	12.1	50.7±22.8 including combined procedures	78.9±32.3 including combined procedures	4 (1.6%)	14 (5.9%)	5(2.1%)	10.1 ± 4.7	1.5 ± 0.4
Cavalier trial^[19]^ (2016) n = 658	Perceval S	10.2±7.8	35.3±12.1	58.4±20.2	5 (0.8)	42 (6.7%)	23 (3.7)	10.24	1.46
Gilmanov^[23]^ (2014) n=133	Predominantly Perceval S	7.5 (4.8-11)	56 (48-72.5)	90 (78-108.5)		3 (2.3%)	2 (1.5)	12	
Pollari^[16]^ (2014) n=133	Perceval S	14±7.4	35±12	71±11	0	5 (6.1%)			
Dalén^[24]^ (2016) n=182	Perceval S	10.6±7.5	41±17	69±23			3 (1.6)		

AV = atrioventricular; AVR=aortic valve replacement; EuroSCORE=European System for Cardiac Operative Risk Evaluation; PV = paravalvular.

Complete heart block requiring permanent pacemaker is a known complication of AVR. This incidence was 3.6% in our study which matches quite well with Santarpino et al.^[22]^ and Flameng et al.^[18]^ but slightly more than Gilmanov et al.^[23]^ (2.3%). Permanent pacemaker requirement in our study was much less than all the other mentioned studies. Early 30-day mortality was 2.1% in our study, which is almost the same as all the other compared studies. In Flameng et al.^[18]^ study, 3 (9.4%) patients died after 6-12 months of surgery. We have shown pre-discharge mean gradient across aortic valve of 12.5 mmHg and its effective orifice area of 1.5 cm^2^. These values correlate well with all the compared studies.

### Limitations

The major limitation of this study is that it was based on data from a single institution and limited number of cases. Moreover, there is the lack of a control group and a randomization in its design. This study showed only early outcomes, and it remains necessary to obtain data documenting long-term performance.

## CONCLUSION

Perceval valve in aortic position is safe and feasible. Sutureless AVR may become the first choice of procedure in the elderly high-risk population. Further follow-up is needed to evaluate the long-term outcome with this bioprosthesis.

**Table:** 

Authors' roles & responsibilities	
SSM	Substantial contributions to the conception or design of the work; or the acquisition, analysis, or interpretation of data for the work; final approval of the version to be published
SL	Substantial contributions to the conception or design of the work; or the acquisition, analysis, or interpretation of data for the work; final approval of the version to be published
ARS	Substantial contributions to the conception or design of the work; or the acquisition, analysis, or interpretation of data for the work; final approval of the version to be published
SC	Substantial contributions to the conception or design of the work; or the acquisition, analysis, or interpretation of data for the work; final approval of the version to be published
TP	Substantial contributions to the conception or design of the work; or the acquisition, analysis, or interpretation of data for the work; final approval of the version to be published
SS	Substantial contributions to the conception or design of the work; or the acquisition, analysis, or interpretation of data for the work; final approval of the version to be published
